# An Integrated Wireless Wearable Sensor System for Posture Recognition and Indoor Localization

**DOI:** 10.3390/s16111825

**Published:** 2016-10-31

**Authors:** Jian Huang, Xiaoqiang Yu, Yuan Wang, Xiling Xiao

**Affiliations:** 1 Key Laboratory of Image Processing and Intelligent Control, School of Automation, Huazhong University of Science and Technology, Wuhan 430074, China; huang_jan@mail.hust.edu.cn (J.H.); yuxiaoqiang@hust.edu.cn (X.Y.); wang.y.bb@m.titech.ac.jp (Y.W.); 2 Department of Rehabilitation, Union Hospital, Tongji Medical College, Huazhong University of Science and Technology, 1277 Jiefang Avenue, Wuhan 430022, China

**Keywords:** indoor localization, posture recognition, wireless sensor system, set-membership filter

## Abstract

In order to provide better monitoring for the elderly or patients, we developed an integrated wireless wearable sensor system that can realize posture recognition and indoor localization in real time. Five designed sensor nodes which are respectively fixed on lower limbs and a standard Kalman filter are used to acquire basic attitude data. After the attitude angles of five body segments (two thighs, two shanks and the waist) are obtained, the pitch angles of the left thigh and waist are used to realize posture recognition. Based on all these attitude angles of body segments, we can also calculate the coordinates of six lower limb joints (two hip joints, two knee joints and two ankle joints). Then, a novel relative localization algorithm based on step length is proposed to realize the indoor localization of the user. Several sparsely distributed active Radio Frequency Identification (RFID) tags are used to correct the accumulative error in the relative localization algorithm and a set-membership filter is applied to realize the data fusion. The experimental results verify the effectiveness of the proposed algorithms.

## 1. Introduction

There are many problems that have arisen due to the fast-aging population all over the world. Among them, health care for and monitoring of the elderly is one of the most important issues that should be addressed. Since more and more elderly people are living alone, a kind of sensor system is urgently needed, which can monitor the posture and location of the elderly. When an emergency happens, their families can obtain timely access to the physical condition and location information of the elderly with the help of this sensor system. For instance, if a monitored old person is found to be lying down but not located on the bed, an alarm should be sent out. To effectively detect this situation, the sensor system has to possess both posture recognition and indoor location abilities. So far, there are plenty of researches on sole posture recognition or indoor localization.

The localization problems exist widely in both the micro and macro applications [1,2]. It is well known that the Global Positioning System (GPS) is one of the most successful localization systems. However, the performance of GPS degrades drastically in an indoor environment [3]. To obtain a robust and accurate indoor localization method, a lot of effective methods have been put forward by researchers. The first class of methods can be categorized into wireless communication-based technologies. So far, there are several wireless technologies being used in the indoor localization, such as WiFi [4,5], Bluetooth [6,7], ZigBee [8,9], RFID (Radio Frequency Identification) [10], and so on. The main purpose of a wireless sensor network (WSN) is to determine the position between a moving target and anchor nodes which are distributed over a geographic area based on the signal strength or transmission [11]. In these methods, plenty of anchor nodes are needed to achieve relatively high accuracy, which increases the total cost of the whole system. With the growing number of nodes, the complexity of the system increases drastically. When the energy of anchor nodes is insufficient, the positioning error of the algorithms based on signal strength will increase quickly. Considering these defects, some algorithms based on Inertial Measurement Units (IMUs) are proposed. Fan et al. proposed an indoor localization method using phone inertial sensors [12]. Gusenbauer et al. also used a mobile phone to realize indoor positioning [13]. They have developed algorithms for reliable detection of steps and heading directions. The main disadvantage of this method is that the phone must be held in a hand, and be pointed in the direction of users’ movement. Jimenez et al. used an INS (Inertial Navigation System)/EKT (Extended Kalman Filter) framework and a foot-mounted IMU to realize indoor localization [14]. Hoflinger et al. presented a wireless Micro- Inertial Measurement Unit to realize localization in indoor areas with sensor data fusion based on Kalman Filter and ZUPT (Zero Velocity Update) [15]. Zhang et al. presented a novel indoor localization and monitoring system based on inertial sensors for emergency responders [16]. They also used the ZUPT method for localization. Some IMUs were attached on different segments to monitor the orientation information of each human body segment. However, they did not give a clear gait or posture recognition method and the localization error is relatively large. In order to overcome the accumulative error, more sensors are used in the indoor localization system. Ultrasonic rangefinder is used to detect the still-phase of the ZUPT method [17]. A three-axis magnetometer is used for heading estimation in [18]. Antonio et al. used a Foot-Mounted IMU and some RFID tags to accurately locate persons indoors [19]. Most of these localization algorithms are based on the ZUPT method. The positioning accuracy of ZUPT method strongly relies on the double integral of the measured acceleration by the inertial sensor. Unfortunately, the accumulative error will increase drastically in the repetitive double integral process [20]. The zero-velocity detection is another key technique of the ZUPT method. That is, the heel strike and heel off should be accurately detected during the user’s walking movement. A wrong zero-velocity detection results in wrong starting and ending time of the double integral.

There are two kinds of approaches often used in posture recognition, including the vision-based approach and approach based on inertial sensors. Boulay et al. proposed an approach to recognise human postures (sitting, standing, bending and lying) in video sequences, which combines a 2D approach with a 3D human model [21]. Le et al. have proposed a method for human posture recognition using a skeleton provided by Kinect device [22]. Yang et al. proposed a portable single-camera gait kinematics analysis system with autonomous knee angle measuring and gait event detection functionality [23]. Diraco et al. presented an active vision system for the automatic detection of falls and the recognition of several postures for elderly homecare applications [24]. The main advantage of vision-based approaches is that they are less intrusive, because the cameras are installed on the building (not worn by users). The disadvantage is that multiple cameras have to be installed in each room. Therefore, the cost is high and the users may be worried about their privacy. Different from the vision-based methods, the methods based on inertial sensors have advantages including the robustness to the ambient light, high precision, easy use and low cost. The disadvantage is also obvious, i.e., inertial sensors have to be worn by the user. Gallagher et al. have presented a technique that computes accurate posture estimates in real-time from inertial and magnetic sensors [25]. In [26], a mobile three-dimensional posture measurement system is developed based on inertial sensors and smart shoes. Harms et al. [27] analyzed the influence of a smart garment on the posture recognition performance used for shoulder and elbow rehabilitation, some garment-attached and skin-attached acceleration sensors were used. Zhang et al. [28] investigated an optimal model selection for posture recognition in home-based healthcare, the tri-axis acceleration signal obtained by a smart phone were used. Gjoreski et al. [29] investigated the impact of accelerometer number and placement on the accuracy of posture recognition. Considering that each sensor modality has its own limitations, some researchers tried to use the fusion of vision and inertial sensor data to improve the accuracy of recognition [30].

It should be noted that most research achievements just focus on a sensor system with sole posture recognition or indoor localization function. Currently, there are not many studies on the integrated sensor systems which combine both of these two functions. Redondi et al. proposed an integrated system based on wireless sensor networks for patient monitoring, localization and tracking [31]. An RF (radio frequency)-based localization algorithm was used to realize indoor localization and a bi-axial accelerometer is used to classify four human movements (prone, supine, standing and walking). Lee et al. used wearable sensors to determine a user’s location and recognize sitting, standing and walking behaviors [32].

In this paper, we designed a wireless sensor node which is used to collect the acceleration, angular velocity and magnetic field strength of a human body segment. Five wireless sensor nodes are respectively fixed on two thighs, two shanks and the waist of the user to obtain the posture information. A standard Kalman filter is used to get more precise posture data. The pitch angles of the thigh and waist are used to realize posture recognition based on a common-used minimum distance classifier. The coordinate values of the lower limb joints are calculated online, and the result is used to compute a one-step vector. We proposed a novel algorithm based on human kinematics to realize the relative indoor localization, which is different from conventional ZUPT methods. Sparsely distributed active RFID tags are used to correct the positioning error, realizing the absolute localization. An ellipsoidal set-membership filter with incomplete observation is applied to achieve the data fusion for enhancing the localization accuracy. The main contribution of this study is to develop a novel wearable sensor system, which combines the functions of posture recognition and indoor localization. A new indoor localization method based on RFID tags and IMUs is proposed, which is better than a conventional method based only on IMU sensors. Compared with the indoor localization method based on wireless technology (e.g., [31]), our proposed system can achieve more accurate localization with fewer anchor nodes. Compared with the dead-reckoning method in [32], our system can recognize more postures besides the advantage of high-precision indoor localization. Also, the zero-velocity detection in the conventional ZUPT method is no longer needed in our proposed system.

The rest of this paper is organized as follows. Section 2 describes the structure and working principle of our integrated wearable sensor system and gives the design detail of our sensor nodes. Section 3 presents the proposed posture recognition algorithm and indoor localization algorithm with a set-membership filter. Section 4 evaluates the proposed algorithms by various experiments. Finally, Section 5 draws the conclusions.

## 2. System Design

### 2.1. System Structure and Working Principle

**Figure 1:**
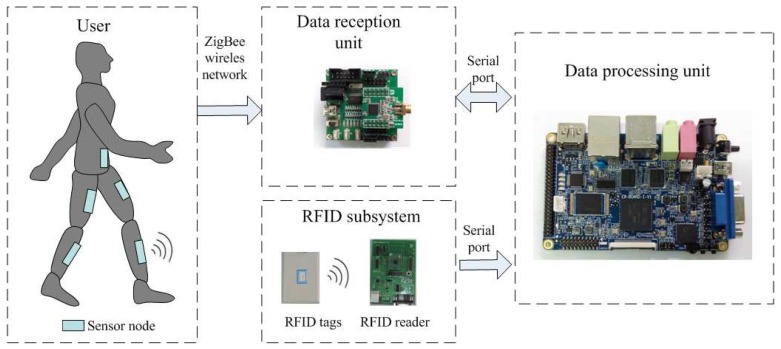
The structure of the integrated wireless wearable sensor system.

**Figure 2:**
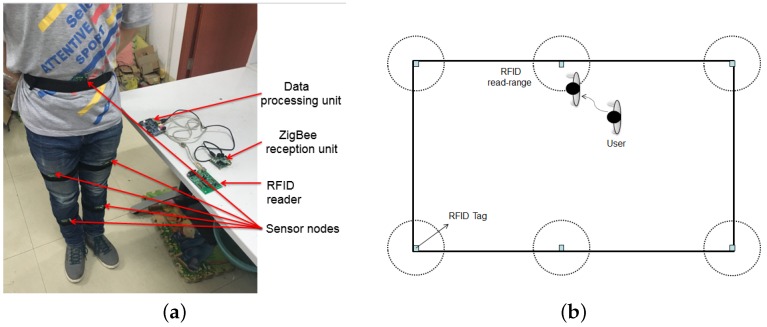
The structure of the whole system. (**a**) The picture of proposed wearable sensor system; (**b**) Work principle of indoor localization corrected by Radio Frequency Identification (RFID) tags.

The structure of the integrated wireless wearable sensor system is shown in Figure 1. The whole system consists of five sensor nodes, a central node for data collection, a data processing unit based on a Samsung Cortex-A8 S5PV210 platform, several active RFID tags and an RFID reader. The sensor nodes are respectively fixed at the waist, both thighs and both shanks of the user (see Figure 2a). The sensor nodes are used to collect the tri-axial acceleration, the angular velocity and the magnetic field strength of the corresponding body parts. A wireless sensor network is formed by the central node and five sensor nodes. The data of each sensor node are periodically sent to the central node in terms of the ZigBee wireless network protocol for a fixed period of time, and the sampling frequency is 20 Hz. After collecting all data from the sensor nodes for one cycle, the central node sends these data to the data processing unit via a USB interface. The posture information of each body part is then extracted from the sensory data by the data processing unit, which can be used to calculate the attitude angles and human joint coordinate values (see the details in the Section 3).

The active RFID tags are deployed at some vital positions of the indoor environment, which are used to correct the localization error of wearable sensors. The RFID reader is connected to the data processing unit through a USB cable, and carried by the user. When the user steps into the read range of an active RFID tag, its unique ID is then recognized by the RFID reader and sent to the data processing unit. The current position of the user can then be calibrated by the preset position of the corresponding RFID tag. The whole process is shown in Figure 2b.

**Figure 3:**
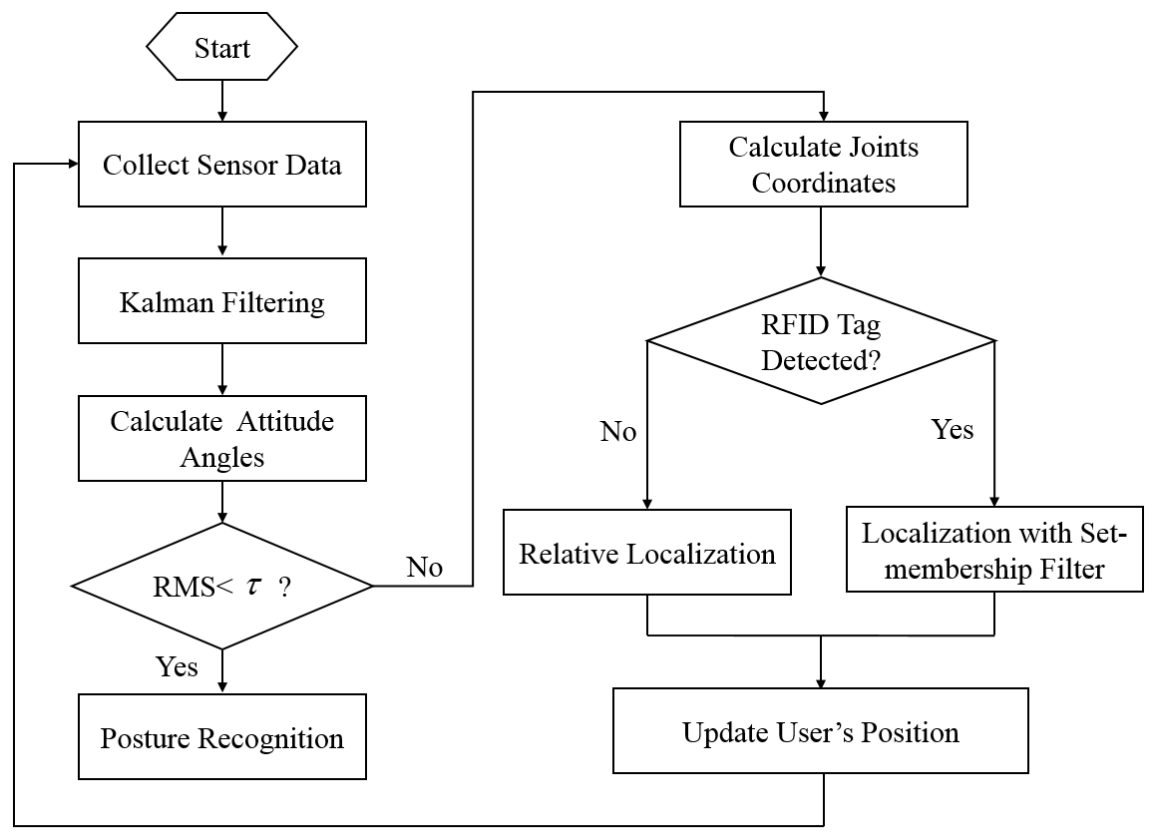
The flow chart of the whole system.

The whole system can be divided into two parts, the posture recognition subsystem and the localization subsystem. When the user is in a static state, the posture recognition algorithm is used to recognize the user’s posture. When the user is in a state of motion, the indoor localization algorithm is used to determine the location of the user. The RMS (root mean square) of the angular velocity of the tri-axial gyroscope at the waist is used to judge if the user is static or not. The RMS is calculated by:

(1) $$\mathrm{RMS}=\sqrt{\omega_{x}^{2}+\omega_{y}^{2}+\omega_{z}^{2}}$$

where, $\omega_{x}$, $\omega_{y}$ and $\omega_{z}$ are the outputs of the tri-axial gyroscope at waist. When the user is static, the RMS is close to zero. Setting a small threshold value *τ*, when the RMS satisfies RMS<τ, the user is then thought to be in a static state. The flow chart of the whole system is shown in Figure 3.

### 2.2. Sensor Node Design

**Figure 4:**
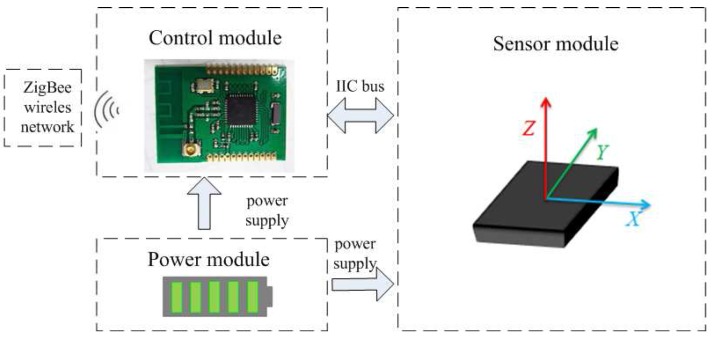
The structure of the sensor node.

In this study, we designed a small-sized and light-weight sensor node which consists of three parts: the control module, the power module and the sensor module (see Figure 4). The Texas instruments’ CC2530 chip was chosen as the control module, which communicates with the sensor module through an IIC-bus protocol to obtain the posture data. The CC2530 enables robust network nodes to be built with very low total bill-of-material costs. Combined with the industry-leading and golden-unit-status ZigBee protocol stack from Texas Instruments, the CC2530F256 provides a robust and complete ZigBee solution. Therefore, we can set up a simple and reliable five-to-one wireless data transmission network by the CC2530.

**Figure 5:**
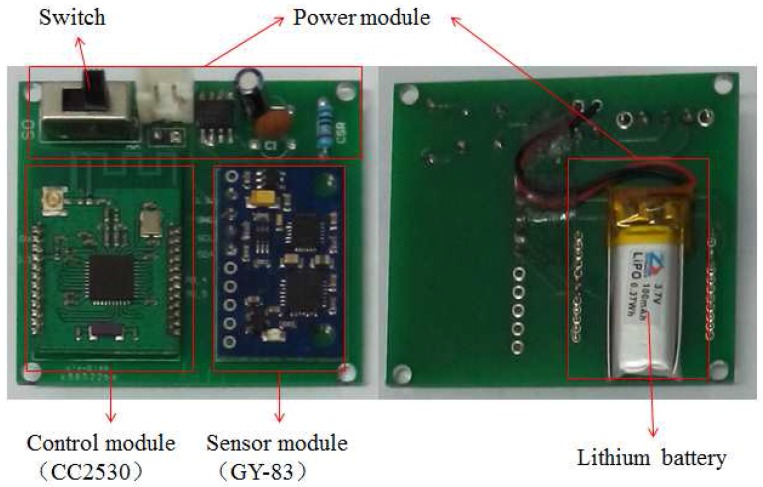
The designed sensor node.

The Micro Electromechanical System (MEMS) sensor GY-83 was chosen as the posture sensor module, which consists of a tri-axial accelerometer, a gyroscope and a magnetometer. The full-range of the acceleration, the angular velocity and the magnetic field intensity are ±4 g, ±500${}^{\circ }$/s and ±1.3 gauss respectively. As for the power module, we use a rechargeable lithium battery and a low dropout regulator (LDO) TPS7333Q to provide a stable voltage of 3.3 V. The whole sensor node is 4.8 cm long and 4.3 cm wide, which is shown in Figure 5.

## 3. Related Algorithm Description

### 3.1. The Calculation of Attitude Angle for Single Sensor Node

**Figure 6:**
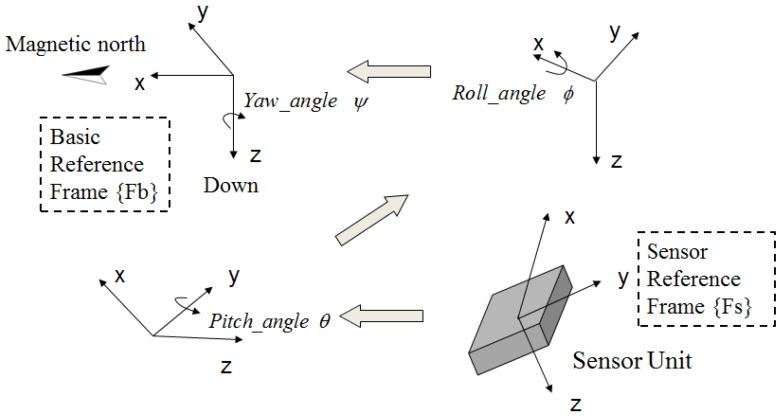
Rotation transformation.

The yaw angle *ψ*, roll angle *ϕ* and pitch angle *θ* are commonly used in inertial navigation to represent the carrier attitude. These angles are referred to as the attitude angles. To calculate the attitude angles, coordinate systems are established first. System {Fb} is defined as the base coordinate system with the *x*-axis pointing to the magnetic north and the *z*-axis to the ground. The *y*-axis of the system {Fb} is determined by the Right-hand rule. We also define a sensor coordinate system {Fs} fixed on the sensor itself (see Figure 6).

Kalman estimation has a quite good effect in data fusion, and it is widely used in various applications including low cost inertial navigation system [33,34,35,36]. Most inertial navigation systems use quaternion as the state variables of Kalman filter. For small wearable sensor systems, considering the computation complexity of the quaternion kalman estimation, Zhu proposed estimation algorithms using the acceleration and magnetic strength as state variables to simplify the calculation [37,38].

For general rotating transformation, the coordinate system that rotates *ϑ* around the vector **n**, can be described by the following rotation transformation matrix:

(2) $$Rot\left(n,\vartheta \right)=\left[\begin{array}{ccc} n_{x}^{2}Vers\vartheta \;+\;C\vartheta & n_{x}n_{y}Vers\vartheta -n_{z}S_{\vartheta } & n_{x}n_{z}Vers\vartheta \;+\;n_{z}S_{\vartheta }\\ n_{x}n_{y}Vers\vartheta \;+\;n_{z}S_{\vartheta } & n_{y}^{2}Vers\vartheta \;+\;C_{\vartheta } & n_{y}n_{z}Vers\vartheta -n_{x}S_{\vartheta }\\ n_{x}n_{z}Vers\vartheta -n_{y}S_{\vartheta } & n_{y}n_{z}Vers\vartheta \;+\;n_{x}S_{\vartheta } & n_{z}^{2}Vers\vartheta \;+\;C_{\vartheta } \end{array}\right]$$

where $Vers\vartheta \;=\;1-\cos\vartheta ,S_{\vartheta }=\sin\vartheta ,C_{\vartheta }=\cos\vartheta$. $n={\left[\begin{array}{ccc} n_{x} & n_{y} & n_{z} \end{array}\right]}^{T}$ denotes the standard orthonormal basis of the general rotation .

Considering the dynamic process of a posture sensor, let us use *t* and t+Δt respectively to denote the start moment and the end moment of a process. Assuming that the period Δt is very small, then we have cosϑ≈1 and sinϑ≈ϑ at time *t*. Thus Equation (1) can be written as:

(3) $$Rot\left(n(t),\vartheta \right)\approx \left[\begin{array}{ccc} 1 & -n_{z}\left(t\right)\vartheta & n_{y}\left(t\right)\vartheta \\ n_{z}\left(t\right)\vartheta & 1 & -n_{x}\left(t\right)\vartheta \\ -n_{y}\left(t\right)\vartheta & n_{x}\left(t\right)\vartheta & 1 \end{array}\right]=\left[\begin{array}{ccc} 1 & -\omega_{z}\left(t\right)\Delta t & \omega_{y}\left(t\right)\Delta t\\ \omega_{z}\left(t\right)\Delta t & 1 & -\omega_{x}\left(t\right)\Delta t\\ -\omega_{y}\left(t\right)\Delta t & \omega_{x}\left(t\right)\Delta t & 1 \end{array}\right]$$

where $\omega_{x}\left(t\right)$, $\omega_{y}\left(t\right)$ and $\omega_{z}\left(t\right)$ are the outputs of the tri-axial gyroscope, which satisfy the follo wing equations:

(4) $$\left\{\begin{array}{c} \omega_{x}\left(t\right)=n_{x}{\left(t\right)\cdot \left(\vartheta /\Delta t\right)|}_{\Delta t\to 0}\\ \omega_{y}\left(t\right)=n_{y}{\left(t\right)\cdot \left(\vartheta /\Delta t\right)|}_{\Delta t\to 0}\\ \omega_{z}\left(t\right)=n_{z}{\left(t\right)\cdot \left(\vartheta /\Delta t\right)|}_{\Delta t\to 0} \end{array}\right.$$

The rotation transformation of posture sensor from time *t* to t+Δt can be expressed by:

(5) $$\left\{\begin{array}{c} {\left[\begin{array}{ccc} g_{x}\left(t+\Delta t\right) & g_{y}\left(t+\Delta t\right) & g_{z}\left(t+\Delta t\right) \end{array}\right]}^{T}=Rot\left(n(t),\vartheta \right){\left[\begin{array}{ccc} g_{x}\left(t\right) & g_{y}\left(t\right) & g_{z}\left(t\right) \end{array}\right]}^{T}\\ {\left[\begin{array}{ccc} H_{x}\left(t+\Delta t\right) & H_{y}\left(t+\Delta t\right) & g_{z}\left(t+\Delta t\right) \end{array}\right]}^{T}=Rot\left(n(t),\vartheta \right){\left[\begin{array}{ccc} H_{x}\left(t\right) & H_{y}\left(t\right) & H_{z}\left(t\right) \end{array}\right]}^{T} \end{array}\right.$$

where ${\left[\begin{array}{ccc} g_{x}\left(t\right) & g_{y}\left(t\right) & g_{z}\left(t\right) \end{array}\right]}^{T}$ is the gravity acceleration vector and ${\left[\begin{array}{ccc} H_{x}\left(t\right) & H_{y}\left(t\right) & H_{z}\left(t\right) \end{array}\right]}^{T}$ is the magnetic field intensity vector in sensor system {Fs}.

For the calculation in the digital processor, the dynamic equations should be discretized. Let *T* denote the sampling period, the dynamic discrete model of Kalman filter is given by:

(6) $$\left\{\begin{array}{c} S(k)=A(k)S(k-1)+W(k)\\ Z(k)=S(k-1)+V(k) \end{array}\right.$$

where **W**(k) and **V**(k) respectively denote the process noise and the observation noise.

The state vector is defined by

(7) $$S={\left[\begin{array}{cccccc} g_{x} & g_{y} & g_{z} & H_{x} & H_{y} & H_{z} \end{array}\right]}^{T}$$

And the observation vector satisfies:

(8) $$Z={\left[\begin{array}{cccccc} a_{x} & a_{y} & a_{z} & h_{x} & h_{y} & h_{z} \end{array}\right]}^{T}$$

where $a_{x}$, $a_{y}$ and $a_{z}$ are the outputs of tri-axial accelerometer. $h_{x}$ , $h_{y}$ and $h_{z}$ are the outputs of the tri-axial accelerometer.

From Equations (2) and (4), the process matrix A(k) at time *k* can be obtained by:

(9) $$A\left(k\right)=\left[\begin{array}{cccccc} 1 & -\omega_{z}\left(k\right)\Delta t & \omega_{y}\left(k\right)\Delta t & 0 & 0 & 0\\ \omega_{z}\left(k\right)\Delta t & 1 & -\omega_{x}\left(k\right)\Delta t & 0 & 0 & 0\\ -\omega_{y}\left(k\right)\Delta t & \omega_{x}\left(k\right)\Delta t & 1 & 0 & 0 & 0\\ 0 & 0 & 0 & 1 & -\omega_{z}\left(k\right)\Delta t & \omega_{y}\left(k\right)\Delta t\\ 0 & 0 & 0 & \omega_{z}\left(k\right)\Delta t & 1 & -\omega_{x}\left(k\right)\Delta t\\ 0 & 0 & 0 & -\omega_{y}\left(k\right)\Delta t & \omega_{x}\left(k\right)\Delta t & 1 \end{array}\right]$$

At each time *k*, the optimal estimation of state variables is calculated from standard Kalman filter procedure and denoted by:

(10) $$\hat{S}\left(k\right)={\left[\begin{array}{cccccc} {\hat{g}}_{x} & {\hat{g}}_{y} & {\hat{g}}_{z} & {\hat{H}}_{x} & {\hat{H}}_{y} & {\hat{H}}_{z} \end{array}\right]}^{T}$$

The rotational transformation matrix from the sensor coordinate system to the base coordinate system is defined as $C_{b}^{s}$. The rotation motion is realized by the following procedure. Firstly, let us rotate system {Fs} around the positive direction of *y*-axis by angle *θ* (its range is −180${}^{\circ }$ to 180${}^{\circ }$). Then rotate it around the positive direction of *x*-axis by angle *ϕ* (its range is −90${}^{\circ }$ to 90${}^{\circ }$). Finally, rotate it around the positive direction of *z*-axis by angle *ψ* (its range is −180${}^{\circ }$ to 180${}^{\circ }$). The whole procedure is shown in Figure 6.

Thus, we have:

(11) $$\begin{array}{c} C_{b}^{s}=Rot\left(y,\theta \right)Rot\left(x,\phi \right)Rot\left(z,\psi \right)\\ =\left[\begin{array}{ccc} \cos\theta_{} & 0 & \sin\theta \\ 0 & 1 & 0\\ -\sin\theta & 0 & \cos\theta \end{array}\right]\cdot \left[\begin{array}{ccc} 1 & 0 & 0\\ 0 & \cos\phi & -\sin\phi \\ 0 & \sin\phi & \cos\phi \end{array}\right]\cdot \left[\begin{array}{ccc} \cos\psi & -\sin\psi & 0\\ \sin\psi & \cos\psi & 0\\ 0 & 0 & 1 \end{array}\right]\;\\ =\left[\begin{array}{ccc} C_{\theta }C_{\psi }\;+\;S_{\theta }S_{\phi }S_{\psi } & -C_{\theta }S_{\psi }+S_{\theta }S_{\phi }C_{\psi } & S_{\theta }C_{\phi }\\ C_{\phi }S_{\psi } & C_{\phi }C_{\psi } & -S_{\phi }\\ -S_{\theta }C_{\psi }+C_{\theta }S_{\phi }S_{\psi } & S_{\theta }S_{\psi }+C_{\theta }S_{\phi }C_{\psi } & C_{\theta }C_{\phi } \end{array}\right] \end{array}$$

where $C_{X}$ and $S_{X}$ respectively represent cosX and sinX.

The optimal estimation of the gravity acceleration vector in the sensor coordinate system is denoted as ${\left[\begin{array}{ccc} {\hat{g}}_{x} & {\hat{g}}_{y} & {\hat{g}}_{z} \end{array}\right]}^{T}$. The optimal estimation of the magnetic field intensity vector is ${\left[\begin{array}{ccc} {\hat{H}}_{x} & {\hat{H}}_{y} & {\hat{H}}_{z} \end{array}\right]}^{T}$ in the sensor system. The different representations of the gravity and geomagnetic intensity in different coordinate systems have the following relationships:

(12) $$\left\{\begin{array}{l} {\left[\begin{array}{ccc} {\hat{g}}_{x} & {\hat{g}}_{y} & {\hat{g}}_{z} \end{array}\right]}^{T}=C_{b}^{s}g^{earth}=C_{b}^{s}{\left[\begin{array}{cc} \begin{array}{cc} 0 & 0 \end{array} & 1 \end{array}\right]}^{T}\;\\ {\left[\begin{array}{ccc} H_{x}^{b} & 0 & H_{z}^{b} \end{array}\right]}^{T}=Rot\left(z,\psi \right){\left[\begin{array}{ccc} H_{x}^{h} & H_{y}^{h} & H_{z}^{h} \end{array}\right]}^{T}\\ {\left[\begin{array}{ccc} H_{x}^{h} & H_{y}^{h} & H_{z}^{h} \end{array}\right]}^{T}=Rot\left(x,-\phi \right)Rot\left(y,-\theta \right){\left[\begin{array}{ccc} {\hat{H}}_{x} & {\hat{H}}_{y} & {\hat{H}}_{z} \end{array}\right]}^{T} \end{array}\right.$$

where ${\left[\begin{array}{ccc} H_{x}^{h} & H_{y}^{h} & H_{z}^{h} \end{array}\right]}^{T}$ is the magnetic field vector in the horizontal plane coordinate system, ${\left[\begin{array}{ccc} H_{x}^{b} & 0 & H_{z}^{b} \end{array}\right]}^{T}$ is the magnetic field vector in the base coordinate system {Fb}.

Form (10) and (11), the yaw angle *ψ*, roll angle *ϕ* and pitch angle *θ* can be calculated as follows:

(13) $$\begin{array}{c} \psi =\left\{\begin{array}{c} \arctan(H_{{}_{y}}^{h}/H_{{}_{x}}^{h})\\ \pi +\arctan(H_{{}_{y}}^{h}/H_{{}_{x}}^{h})\\ -\pi +\arctan(H_{{}_{y}}^{h}/H_{{}_{x}}^{h})\\ \pi /2\\ -\pi /2 \end{array}\;\;\;\begin{array}{c} H_{{}_{x}}^{h}>0\\ H_{{}_{y}}^{h}>0\;and\;H_{{}_{x}}^{h}<0\\ H_{{}_{y}}^{h}<0\;and\;H_{{}_{x}}^{g}<0\\ H_{{}_{y}}^{h}>0\;and\;H_{{}_{x}}^{h}=0\\ H_{{}_{y}}^{h}<0\;and\;H_{{}_{x}}^{h}=0 \end{array}\right. \end{array}$$

(14) $$\begin{array}{c} \phi =-\arctan({\hat{g}}_{y}/\sqrt{{\hat{g}}_{x}^{2}+{\hat{g}}_{z}^{2}}) \end{array}$$

(15) $$\begin{array}{c} \theta =\left\{\begin{array}{c} \arctan({\hat{g}}_{x}/{\hat{g}}_{z})\\ \pi +\arctan({\hat{g}}_{x}/{\hat{g}}_{z})\\ -\pi +\arctan({\hat{g}}_{x}/{\hat{g}}_{z})\\ \pi /2\\ -\pi /2 \end{array}\;\;\;\begin{array}{c} {\hat{g}}_{z}>0\\ {\hat{g}}_{x}>0\;and\;{\hat{g}}_{z}<0\\ {\hat{g}}_{x}<0\;and\;{\hat{g}}_{z}<0\\ {\hat{g}}_{x}>0\;and\;{\hat{g}}_{z}=0\\ {\hat{g}}_{x}<0\;and\;{\hat{g}}_{z}=0 \end{array}\right. \end{array}$$

### 3.2. Posture Recognition

**Figure 7:**
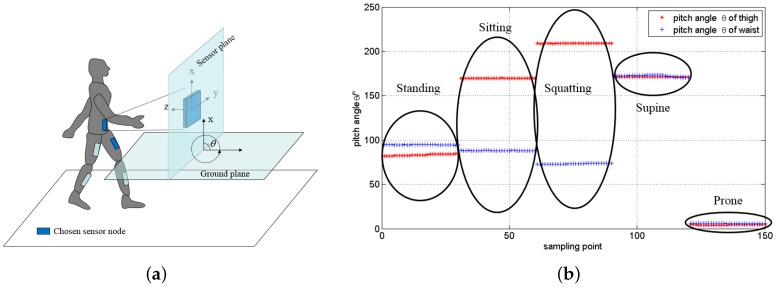
The features selection of posture recognition. (**a**) The illustration of pitch angle; (**b**) The pitch angles of the left thigh and waist in each posture.

The attitude angles of the thighs, shanks and waist can be represented by the yaw angle *ψ*, roll angle *ϕ* and pitch angle *θ* of the sensors on corresponding body segments, which are calculated by the Equations (13)–(15). To carry out efficient and real-time posture recognition, first we need to extract some of the most important features. As shown in Figure 7a, the pitch angle *θ* can represent the tilt angle between the body segment and the flat ground. For simplicity of computation, here we assume that the range of pitch angle is within [0${}^{\circ }$, 360${}^{\circ }$]. The pitch angles of left thigh and waist are chosen as the features to distinguish the five postures (sitting, standing, squatting, supine and prone) in daily life. We collected 30 sampling points of each posture respectively, and Figure 7b gives the pitch angle scatter diagram. As shown in Figure 7b, the difference of pitch angles between each two postures is very obvious. Thus, the five postures can be distinguished easily by using these two features.

The common-used minimum distance classifier is used to recognize the five postures. Let $m_{k}=\left[\theta_{t}^{k},\theta_{w}^{k}\right]\left(k=1,2,\ldots ,5\right)$ denote the mean vector of *k*-th posture. Let 1, 2, 3, 4 and 5 respectively denote standing, sitting, squatting, supine and prone posture. $\theta_{t}^{k}$ is the pitch angle of left thigh of *k*-th posture, and $\theta_{w}^{k}$ is the pitch angle of waist of *k*-th posture. To realize posture recognition, firstly *K* training samples of each posture are used to estimate the five mean vector $m_{k}$ by the following equation:

(16) $$m_{k}=\frac{1}{K}\sum_{i=1}^{K}y_{{}_{i}}^{k}\left(k=1,2\ldots ,5\right)$$

where *K* = 50, $y_{i}^{k}$ is the *i*-th training sample of *k*-th posture.

Then for a new sampling point $y_{j}=\left[\theta_{t}^{j},\theta_{w}^{j}\right]$ , it is classified to $m_{k}$ if its Euclidean distance to $m_{k}$ is smaller than those to all other classes:

(17) $$y_{j}\in m_{k}ifd\left(y_{j},m_{k}\right)=\min\left\{d\left(y_{j},m_{i}\right)i=1,2\ldots ,5\right\}$$

where $d\left(y_{j},m_{i}\right)=\sqrt{{\left(\theta_{t}^{j}-\theta_{t}^{i}\right)}^{2}+{\left(\theta_{w}^{j}-\theta_{w}^{i}\right)}^{2}},\left(i=1,2,\ldots ,5\right)$.

### 3.3. Localization Algorithm Based on Inertial Navigation

#### 3.3.1. The Calculation of Joints Coordinates

**Figure 8:**
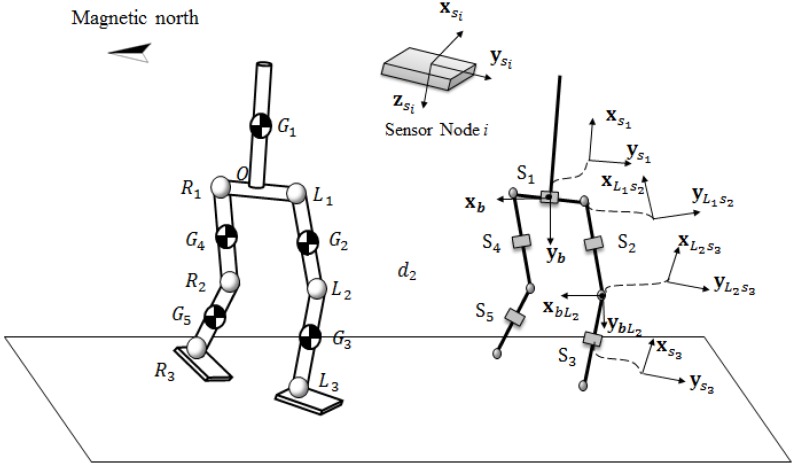
Rotation transformation.

To calculate the coordinates of human body joints, we first define several important coordinate systems (see Figure 8). System {b} is a base reference coordinate system which is fixed at the midpoint of the two hip joints with the same direction as {Fb} given in Section 3.1. The origin of this coordinate system is the midpoint of user’s two hip joints. System {$s_{i}$} is a sensor coordinate system fixed on sensor *i* (i=1,2,…,5) with its *z*-axis perpendicular to the sensor surface and *y*-axis parallel to the sensor surface. For each hip or knee joint point, there is a coordinate system fixed on it with the same orientation as system {b} and a coordinate system fixed on it with the same orientation as the sensor coordinate system {$s_{i}$}. For example, system {$bL_{2}$} is fixed on the left knee joint with the z-axis pointing downwards and the x-axis pointing to the magnetic north. The origin of coordinate system {$L_{2}$$s_{3}$} is the left knee joint, whose orientation is the same as the sensor coordinate system {$s_{3}$}.

From Equations (13)–(15), we can get each sensor’s yaw angle $\psi_{i}$, roll angle $\phi_{i}$ and pitch angle $\theta_{i}$ in the sensor coordinate system $\left\{s_{i}\right\}$. $C_{s1}^{b}$ is the rotation transformation matrix which describes the rotation from coordinate system {b} to sensor coordinate system $\left\{s_{i}\right\}$ and can be given by:

(18) $$C_{si}^{b}=Rot\left(z,-\psi_{i}\right)Rot\left(x,-\phi_{i}\right)Rot\left(y,-\theta_{i}\right)={\left(C_{b}^{si}\right)}^{-1}\begin{array}{cc} , & \left(i=1,2,\ldots ,5\right) \end{array}$$

Then we can compute the coordinate values of all joint points by:

(19) $$\left\{\begin{array}{c} X_{L1}^{b}=C_{s1}^{b}X_{L1}^{s1},\left(X_{L1}^{s1}={\left[\begin{array}{ccc} 0 & l_{waist}/2 & 0 \end{array}\right]}^{T}\right)\\ X_{L2}^{b}=C_{L1s2}^{bL1}X_{L2}^{L1s2}+X_{L1}^{b},\left(X_{L2}^{L1s2}={\left[\begin{array}{ccc} -l_{thigh} & 0 & 0 \end{array}\right]}^{T},\;C_{L1s2}^{bL1}=C_{s2}^{b}\right)\\ X_{L3}^{b}=C_{L2s3}^{bL2}X_{L3}^{L2s3}+X_{L2}^{b},\left(X_{L3}^{L2s3}={\left[\begin{array}{ccc} -l_{shank} & 0 & 0 \end{array}\right]}^{T},\;C_{L21s3}^{bL2}=C_{s3}^{b}\right)\\ X_{R1}^{b}=C_{s1}^{b}X_{R1}^{s1},\left(X_{R1}^{s1}={\left[\begin{array}{ccc} 0 & -l_{waist}/2 & 0 \end{array}\right]}^{T}\right)\\ \begin{array}{c} X_{R2}^{b}=C_{R1s4}^{bR1}X_{R2}^{R1s4}+X_{R1}^{b},\left(X_{R2}^{R1s4}={\left[\begin{array}{ccc} -l_{thigh} & 0 & 0 \end{array}\right]}^{T},\;C_{R1s4}^{bR1}=C_{s4}^{b}\right)\;\\ X_{R3}^{b}=C_{R2s5}^{bR2}X_{R3}^{R2s5}+X_{R2}^{b},\left(X_{R3}^{s5}={\left[\begin{array}{ccc} -l_{shank} & 0 & 0 \end{array}\right]}^{T},\;C_{R2s5}^{bR2}=C_{s5}^{b}\right) \end{array} \end{array}\right.$$

where $X_{L1}^{b},X_{L2}^{b},X_{L3}^{b}$ are the coordinate values of left hip, knee, ankle joint points in the base coordinate system {b}. $X_{R1}^{b},X_{R2}^{b},X_{R3}^{b}$ are the coordinate values of the right hip, knee, ankle joint points in the base coordinate system {b}. $l_{waist}$ is the length of user’s waist. $l_{thigh}$ denotes the length of user’s thigh. $l_{shank}$ denotes the length of user’s shank. $X_{L1}^{s1}$ is the coordinate value of left hip joint in system $\left\{s_{1}\right\}$. $X_{L2}^{L1s2}$ is the coordinate value of left knee joint in system $\left\{L_{1}s_{1}\right\}$, and $X_{L3}^{L2s3},X_{R1}^{s1},X_{R2}^{R1s4},X_{R3}^{s5}$ are similarly defined. Thus, the user’s gait is well described by all the joint points in the base reference coordinate system.

#### 3.3.2. Relative Localization Algorithm Based on Step Length

In this subsection, we propose a relative localization algorithm based on the step length. From Equation (19) in Section 3.3.1, the coordinate values in the base coordinate system {b} of the right ankle joint and left ankle joint can be calculated, which are respectively denoted by $X_{R3}^{b}$ and $X_{L3}^{b}$. The length of one step is the distance between the right ankle and the left ankle, which can be calculated by $\left|X_{R3}^{b}-X_{L3}^{b}\right|$.

The recognition of one step is the most important problem in the relative localization. Let *β* denote the angle between the two thighs, then we have:

(20) $$\cos\beta =\frac{\langle C_{s2}^{bL1}X_{L2}^{s2},C_{s4}^{bR1}X_{R2}^{s4}\rangle }{\left|C_{s2}^{bL1}X_{L2}^{s2}\right|\left|C_{s4}^{bR1}X_{R2}^{s4}\right|}$$

**Figure 9:**
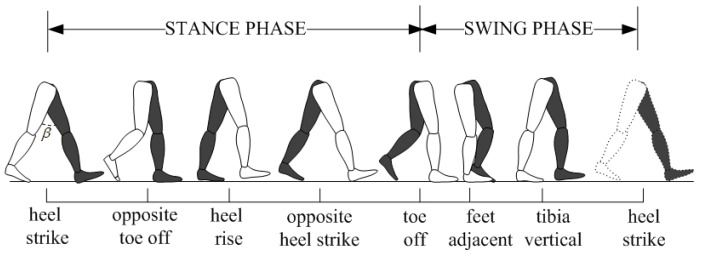
Typical normal gait cycle.

From the research in [39], we know that in a gait cycle the angle *β* will increase to reach a maximum value and then decreases (see Figure 9). One step is formed when the front heel touches the ground while the rear heel is going to be lifted. The angle *β* reaches the maximum value at this moment. If the sensory data at this moment are recorded, the one-step vector can then be obtained.

Since only the 2D ground coordinates are needed in the indoor localization, we just focus on the *x*-axis and *y*-axis coordinate. Let $X_{front}^{b}={\left[\begin{array}{cc} x_{front} & y_{front} \end{array}\right]}^{T}$ denote the coordinate of front ankle joint and $X_{rear}^{b}={\left[\begin{array}{cc} x_{rear} & y_{rear} \end{array}\right]}^{T}$ denote the coordinate of rear ankle joint, respectively. Then the one-step vector can be represented by:

(21) $$L^{b}=X_{front}^{b}-X_{rear}^{b}={\left[\begin{array}{cc} x_{front}-x_{rear} & y_{front}-y_{rear} \end{array}\right]}^{T}$$

**Figure 10:**
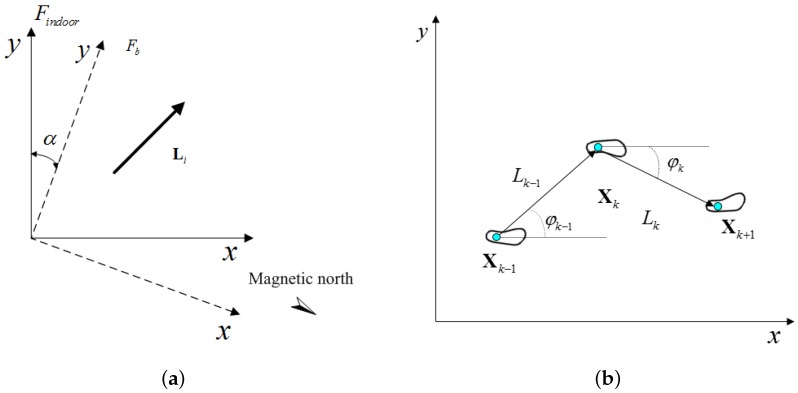
The coordinate definition of the indoor localization subsystem. (**a**) Indoor coordinate system and base coordinate system.; (**b**) Updating of localization algorithm.

After walking for *n* steps, the displacement of user is calculated by $D_{n}=\sum_{i=1}^{n}L_{i}^{b}$, where the $L_{i}^{b}$ represents the *i*-th one-step vector. It is worth noting that the displacement is calculated in the base coordinate system {b}. There is an indoor coordinate system established for the indoor localization. The *x*-axis of base coordinate system is pointing at magnetic north, but the *x*-axis of indoor coordinate system is determined by the building. There is an angle between the base coordinate system and the indoor coordinate system (see Figure 10a), which is denoted by *α*. The rotational transformation matrix from the indoor coordinate system to the base coordinate system is calculated by:

(22) $$C_{b}^{indoor}=\left[\begin{array}{cc} \cos\alpha & -\sin\alpha \\ \sin\alpha & \cos\alpha \end{array}\right]$$

(23) $$L_{i}^{indoor}=C_{b}^{indoor}L_{i}^{b}$$

#### 3.3.3. Set-Membership Filter Algorithm with Incomplete Observation

Note that there is a small error in the measurement of each one-step vector using the proposed wearable sensor system. With the number of steps increasing, the cumulative error increases quickly. This results in larger and larger positioning error, which is not allowable in the localization.

To solve this problem, we use fixed-point tags for error correction. Some active RFID tags are installed in vital positions. When a user walks in the area of a RFID tag, the RFID reader carried by the user can recognize the ID of the RFID tag. Once the RFID reader finds a tag, a data fusion algorithm is needed to fuse the localization data from the relative localization algorithm and the fixed localization data from the RFID tag. A sub-optimal Kalman filter is used in [40], which has the limitation that the noise must be white Gaussian noise. The Kalman filter has a poor performance for non-Gaussian noises [41]. Assuming that the processing and measurement noises are unknown-but-bounded (UBB), an ellipsoidal set-membership filter is applied as follows.

First, the system model of our localization is established as:

(24) $$\left\{\begin{array}{c} X_{k}=F\left(X_{k-1},L_{k-1},\varphi_{k-1}\right)+w_{k-1}\\ Z_{k}=\Gamma_{k}\left({\mathrm{HX}}_{k+1|k}+v_{k}\right) \end{array}\right.$$

where $X_{k}$ is the position state variable vector which is determined by $X_{k}={\left[\begin{array}{cc} x_{k} & y_{k} \end{array}\right]}^{T}$. $x_{k}$ is the *x*-axis coordinate value and $y_{k}$ is the *y*-axis coordinate value of user in the indoor coordinate system. $X_{k}$ and $X_{k-1}$ respectively denote the *k*-th and (k−1)-th position state. $Z_{k}$ is the observation vector. $w_{k}$ and $v_{k}$ respectively denote the process noise and observation noise. H is the observation matrix. $\left\{\Gamma_{k}\right\}$ is an unknown binary sequence composed of 0’s and 1’s. $Z_{k}$ is available if $\Gamma_{k}=1$ while it is missing if $\Gamma_{k}=0$ . $F\left(•\right)$ is the nonlinear rule between $X_{k}$ and $X_{k-1}$, which satisfies:

(25) $$F\left(X_{k-1},L_{k-1},\varphi_{k-1}\right)=\left[\begin{array}{c} x_{k-1}+L_{k-1}\cdot \cos\varphi_{k-1}\\ y_{k-1}+L_{k-1}\cdot \sin\varphi_{k-1} \end{array}\right]$$

where $L_{k-1}$ denotes the (k−1)-th step length. $\varphi_{k-1}$ is the direction angle between the (k−1)-th step vector and the *x*-axis of indoor coordinate system(see Figure 10b).

The description of an ellipsoid is given by a set:

(26) $$\Omega =\left\{x:{\left(x-a\right)}^{T}P^{-1}\left(x-a\right)\leq \sigma^{2}\right\}$$

where *a* is the center of the ellipsoid. *x* is an arbitrary possible value within the ellipsoid, and P is a positive definite matrix that determines the shape of the ellipsoid. *σ* is not a physically interpretable measure of the size of the ellipsoid. It has been noted in [42] that *σ* is usually considered as a measure of optimality in ellipsoid set-membership filter. In the following, we write the ellipsoid as $\Omega \left(a,P,\sigma \right)$.

In the set-membership framework, the process noise can be summarized as the unknown-but-bounded (UBB) noises which belong to the given set:

(27) $$W_{k}=\left\{w_{k}:w_{k}^{T}Q_{k}^{-1}w_{k}\leq \sigma_{w}^{2}\right\}$$

where $Q_{k}$ is the known positive matrix and $\sigma_{k}$ is a known positive scalar which represents the upper bound of the process noise.

The observation noise $v_{k}$ belongs to:

(28) $$V_{k}=\left\{v_{k}:v_{k}^{T}v_{k}\leq \gamma^{2}\right\}$$

where *γ* is also a known positive scalar which represents the upper bound of the observation noise.

The initial state $X_{0}$ belongs to a given set $\Omega \left({\hat{X}}_{0},P_{0},\sigma_{0}\right)$.

The first step of the set-membership filter is time updating. A predictive value is obtained after time updating. Assuming that the state vector satisfies $X\in \Omega \left({\hat{X}}_{k},P_{k},\sigma_{k}\right)$, and the prediction ellipsoid containing the state at time is defined as $X\in \Omega \left(X_{k+1|k},P_{k+1|k},\sigma_{k+1|k}\right)$, then we have:

(29) $$X_{k+1|k}=F_{k}{\hat{X}}_{k}$$

(30) $$P_{k+1|k}=\left(1+p_{k}\right)F_{k}P_{k}F_{k}^{T}+\left(1+p_{k}^{-1}\right)\frac{\sigma_{w}^{2}}{\sigma_{k}^{2}}Q_{k}$$

(31) $$\sigma_{k+1|k}=\sigma_{k}$$

where

(32) $$F_{k}=\frac{\partial F({\hat{X}}_{k},L_{k},\alpha_{k})}{\partial {\hat{X}}_{k}}$$

Equations (34)–(36) are similar to those presented in [43], and the method of selecting $p_{k}$ can be found in [44], which can be concluded as follows: If $p_{k}$ satisfies

(33) $$p_{k}=\frac{\sigma_{w}}{\sigma {}_{k}}\sqrt{\frac{tr(Q_{k})}{tr(F_{k}P_{k}F_{k})}}$$

then the trace of matrix $P_{k+1|k}$ achieves its minimum.

**Figure 11:**
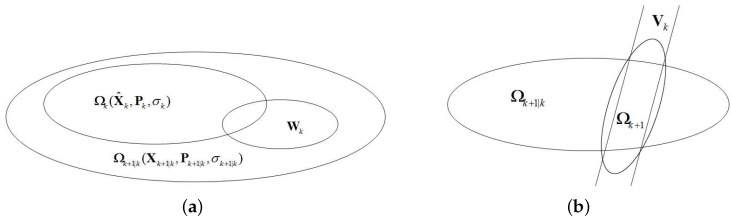
The process of time updating and observation updating without observation missing. (**a**) The process of time updating; (**b**) Observation updating without observation missing.

The whole process of time updating is shown in Figure 11a.

The second step of our filter is the observation updating. Considering the possible loss of measurements, the observation can be categorized into two cases: the observation is available and the observation is missing.

$\Omega \left({\hat{X}}_{k+1},P_{k+1},\sigma_{k+1}\right)$ is defined as the final estimated ellipsoid of our set-membership filter. If there is an observation, then we have:

(34) $$P_{k+1}=\left(I-K_{k+1}H\right)P_{k+1|k}$$

(35) $${\hat{X}}_{k+1}=X_{k+1|k}+K_{k+1}e_{k+1}$$

(36) $$\sigma_{k+1}^{2}=\sigma_{k+1|k}^{2}+q_{k+1}\gamma^{2}-q_{k+1}e_{k+1}^{T}S_{k+1}^{-1}e_{k+1}$$

where

(37) $$S_{k+1}=I+q_{k+1}{\mathrm{HP}}_{k+1|k}H^{T}$$

(38) $$e_{k+1}=Z_{k+1}-{\mathrm{HX}}_{k+1|k}$$

(39) $$K_{k+1}=P_{k+1|k}H^{T}{\left(\frac{1}{q_{k+1}}I+{\mathrm{HP}}_{k+1|k}H^{T}\right)}^{-1}$$

$q_{k+1}$ is a parameter that determines the property of the outer bounding ellipsoid $\Omega \left({\hat{X}}_{k+1},P_{k+1},\sigma_{k+1}\right)$. The method of selecting $q_{k+1}$ has been discussed in [45]. If $q_{k+1}$ satisfies:

(40) $$q_{k+1}=\left\{\begin{array}{cc} 0 & \left|\right|e_{k+1}\left|\right|\leq \gamma \\ \left(1/g_{k+1}\right)\left(e_{k+1}/\gamma -1\right) & \left|\right|e_{k+1}\left|\right|>\gamma \end{array}\right.$$

where $g_{k+1}$ is the maximum singular value of $P_{k+1|k}$, then $\sigma_{k+1}^{2}$ achieves its minimum.

The whole process of observation updating without observation missing is concluded in Figure 11b.

When the observation is missing, we directly choose the ellipsoid $\Omega_{k+1|k}$ as $\Omega_{k+1|}$, that is:

(41) $$P_{k+1}=P_{k+1|k}$$

(42) $${\hat{X}}_{k+1}=X_{k+1|k}$$

(43) $$\sigma_{k+1}=\sigma_{k+1|k}$$

The whole set-membership filter with incomplete observation algorithm is concluded as Algorithm 1.

**Algorithm 1:** Set-membership filter with incomplete observation **Require:**
${\hat{X}}_{k},F_{k},\sigma_{k},\sigma_{w},P_{k},\Gamma_{k+1},\gamma$   1: Calculate $X_{k+1|k}$ from Equation (29)   2: Select the parameter $p_{k}$ from Equation (33)   3: Calculate $\sigma_{k+1|k}$ from Equation (31), calculate $P_{k+1|k}$ from Equation (30)   4: **if**
$\Gamma_{k+1}=1$
**then**   5:  Select the parameter $q_{k}$ from Equation (40)   6:  Calculate ${\hat{X}}_{k+1}$ from Equation (35), calculate $P_{k+1}$ from Equation (34), calculate $\sigma_{k+1}$ from Equation (36)   7: **else**   8:  Calculate ${\hat{X}}_{k+1}$ from Equation (42) , calculate $P_{k+1}$ from Equation (41), calculate $\sigma_{k+1}$ from Equation (43)   9: **end if**  10: **return**
${\hat{X}}_{k+1},P_{k+1},\sigma_{k+1}$

## 4. Experiments Results and Discussion

### 4.1. Posture Recognition Using Wireless Wearable Sensors System

Four male subjects took part in the experiments voluntarily. The physical parameters of subject A (167 cm, 60 kg), subject B (178 cm, 65 kg), subject C (168 cm, 62 kg) and subject D (168 cm, 75 kg) are shown in Table 1.

**Figure 12:**
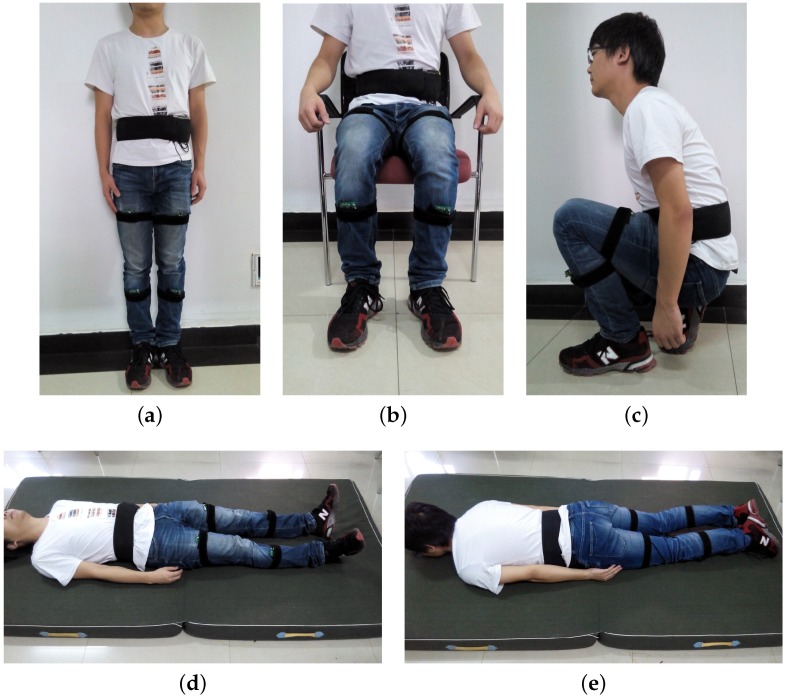
The five postures of proposed posture recognition algorithm. (**a**) Standing posture; (**b**) Sitting posture; (**c**) Squatting posture; (**d**) Supine posture.; (**e**) Prone posture.

Each subject was asked to wear the sensor system and then keep standing, sitting, squatting, supine and prone for a given period of time, respectively. The five postures are shown in Figure 12. Using the method mentioned in Section 3.2, we first established the mean vector of each posture with a number of training data. Then the subject walked freely and kept a certain posture for a short time suddenly, so that the posture recognition algorithm could be used to distinguish the static posture at the same time. Each subject repeated 50 experimental trials for each posture, the success rates of posture recognition are shown in Table 2. Compared with the posture recognition method in [31], in which only an accelerometer was used, more postures are recognized because we combine the posture information of two body segments (the left thigh and waist).

### 4.2. One-Step Vector Measurement Experiments

**Figure 13:**
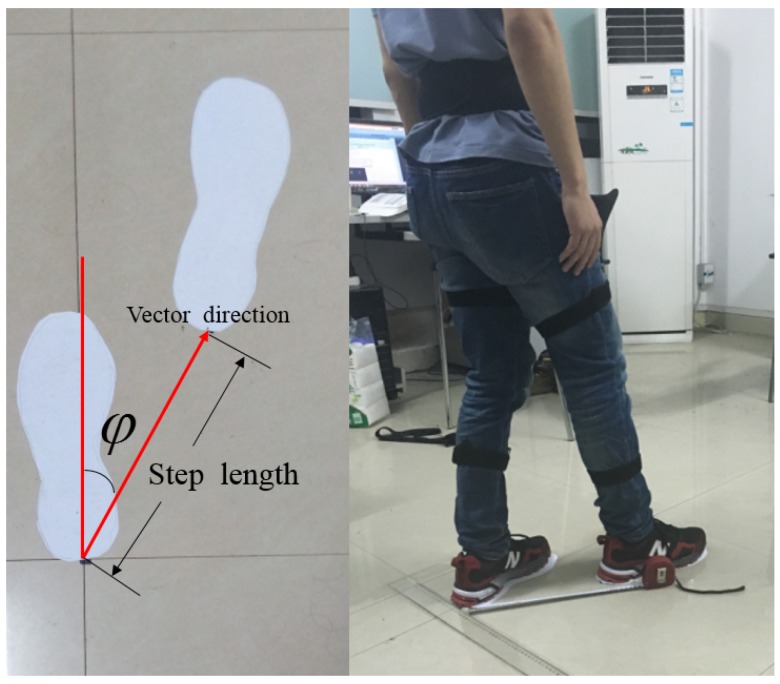
The setup of one-step experiments.

**Figure 14:**
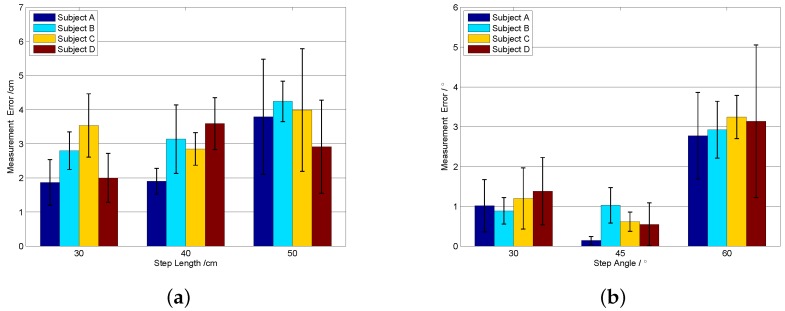
The mean (error bar) and standard deviation (black lines on the error bar) of the measurement error per subject according to the step length and step angle. (**a**) The measurement error per subject according to the step length; (**b**) The measurement per subject according to the step angle.

Every step length calculation is the basis for the indoor localization algorithms. Before the localization experiments, one-step length experiments were conducted to evaluate the performance of the proposed sensor system. Each subject was asked to take a step with different length and towards different directions. The measurement results of the ruler are used as the reference values. The experimental setup is shown in Figure 13. The footprints are marked on the floor, and subject was asked to stand just above the footprints. The step length and angle *φ* are recorded at the same time. For each different step length and angle, 20 repeated experiments were carried out by each subject. Figure 14 gives the measurement error bar graph.

As shown in Figure 14, the mean measurement error of one-step length is smaller than 5 cm, and the maximum measurement error is smaller than 6 cm. The mean measure error of the one-step angle is smaller than 4${}^{\circ }$, and the maximum measurement error is smaller than 6${}^{\circ }$. These errors are acceptable considering the interference of ambient field and the measurement error of the ruler. The experimental results ensure the feasibility of the proposed indoor localization algorithms based on the one-step vector.

### 4.3. Indoor Localization Experiments

#### 4.3.1. Description of Experiments

**Figure 15:**
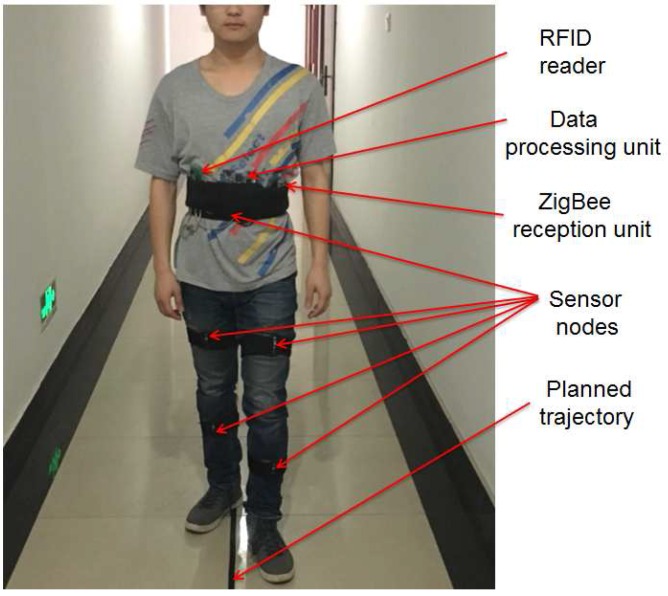
Wearable sensor system for posture recognition and indoor localization.

**Figure 16:**
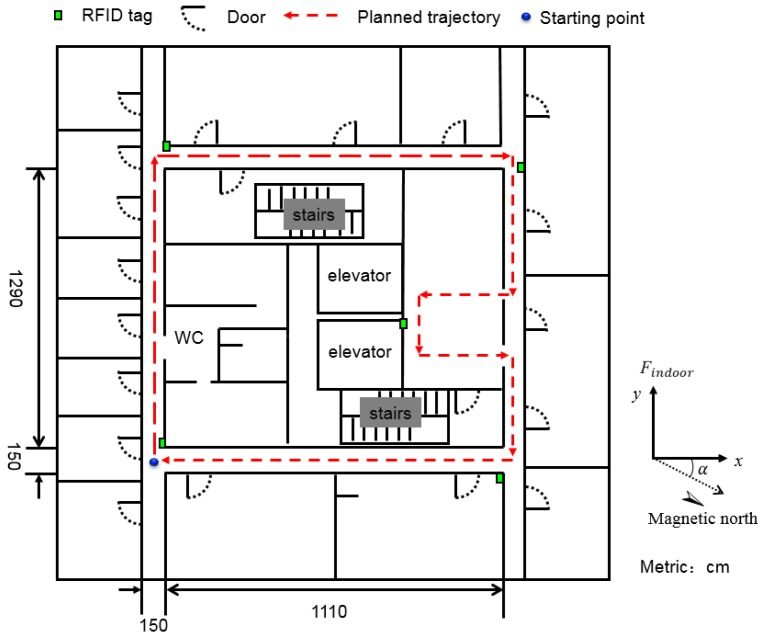
The ichnography of indoor localization environment.

The same four subjects wearing the sensor system took part in the experiments. The appearance of a subject wearing the sensor system is shown in Figure 15. The ichnography of experiment environment is shown in Figure 16. The subjects were asked to walk along the red dashed line which is marked in the ichnography. During the subjects’ walking, the posture data were also measured online. The data processing unit calculated the coordinate value of every step at the same time, so that the indoor localization can be realized simultaneously based on these data.

Considering the characteristics of the planned trajectory, we placed four RFID tags at the four corners and a RFID tag near the elevator (see Figure 16). The coordinate of each RFID tag was saved in the program which is running in the data processing unit. When the user walked into the read-range of the RFID tag, its coordinate value was then used to correct the error of localization. It is worth noting that the read-range of the RFID tag can affect the accuracy of localization. If the range is too big, the error will be relatively large. Meanwhile, the RFID tag may not be detected if the read-range is set too small. Thus, the read-range should be set at a moderate value. In our experiments, the read-range of the RFID tag is empirically set as 1 m.

#### 4.3.2. Experiments on Different Subjects

**Figure 17:**
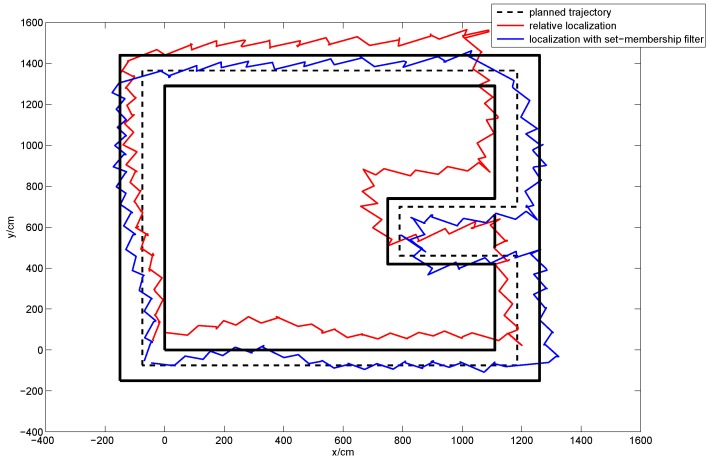
The average trajectory curves of subject A walking with normal steps.

**Figure 18:**
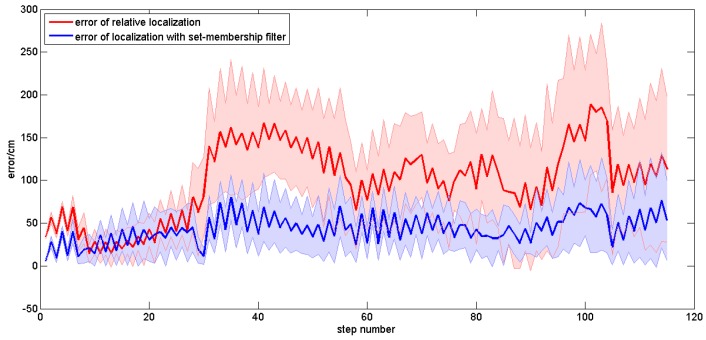
The mean error curves of Subject A walking with normal steps.

**Figure 19:**
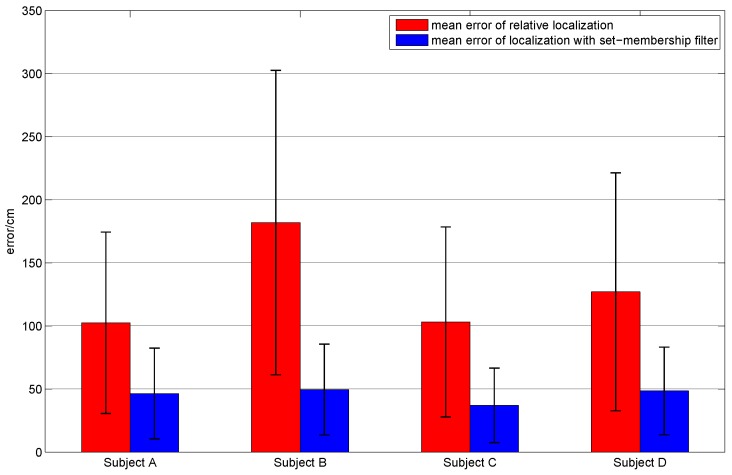
The mean (error bar) and standard deviation (black lines on the error bar) of the localization error per subject using the relative localization algorithm and the proposed algorithm.

In order to evaluate the applicability of our method in different people, plentiful repeated experiments were carried out by the four subjects. Each subject was asked to walk along the planned trajectory 10 times. In order to compare the performances of the sole relative localization algorithm and the localization algorithm with set-membership filter, we plotted the trajectories of these two localization algorithms in one figure. Figure 17 shows the average trajectory of the subject A obtained using our localization approach over 10 repetitions of the experiment, and Figure 18 gives the mean error curves and the standard deviation. An error bar graph is also presented to compare the indoor localization experiment results of the four subjects (see Figure 19).

From Figure 19, we can draw the conclusion that the performance of the localization algorithm with the set-membership filter is excellent and stable. It is observed that the mean error of the sole relative localization is relatively large and it presents different performances for different subjects. Compared with the sole relative localization algorithm, the mean error of localization with the set-membership filter is much smaller. Note that the mean error is less than 50 cm, which is smaller than the result (approximately 1.5 m) proposed in [12,19], and is much better than the result (about 2–3 m) in [31].

#### 4.3.3. Experiments Regarding Different Ways of Walking

**Figure 20:**
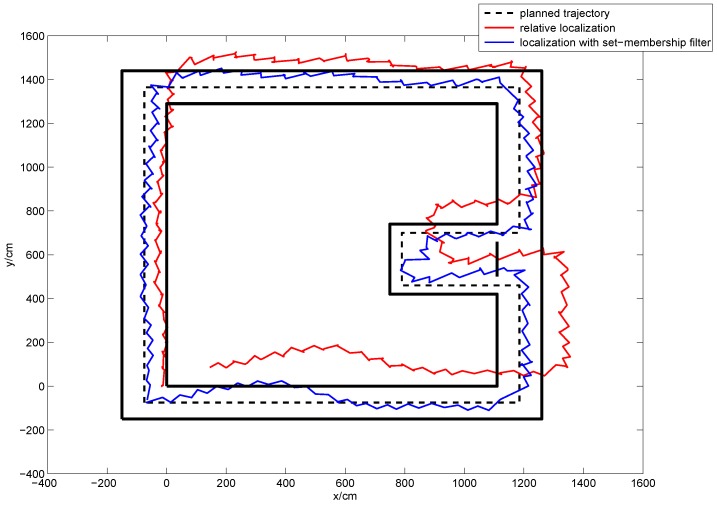
The average trajectory curves of subject A walking with small steps.

**Figure 21:**
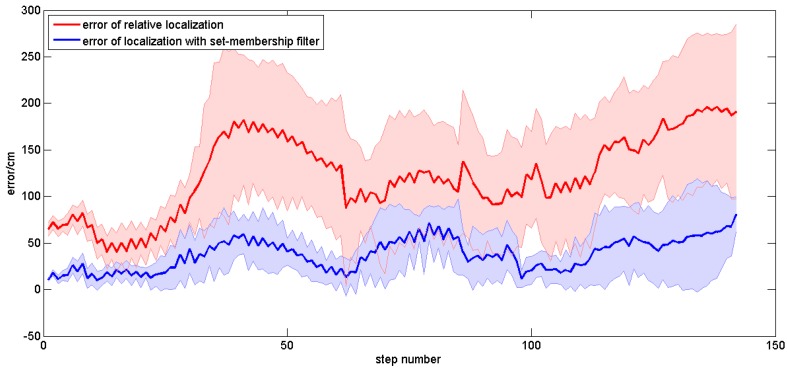
The mean error curves of Subject A walking with small steps.

**Figure 22:**
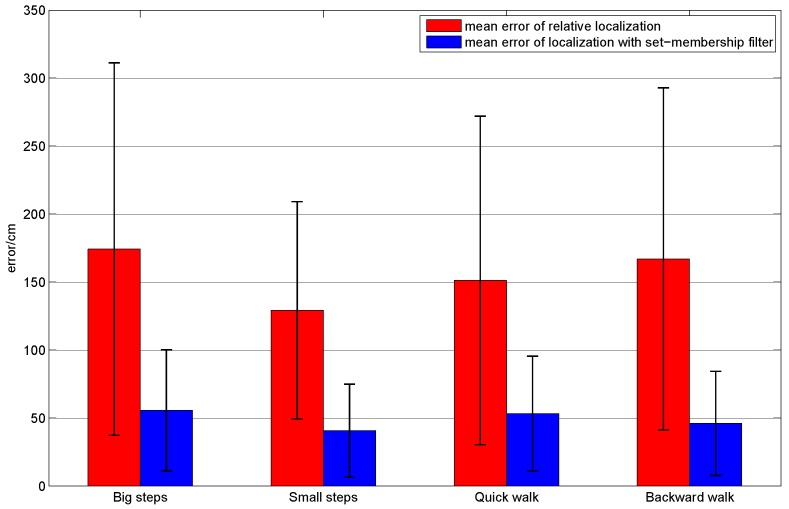
The mean (error bar) and standard deviation (black lines on the error bar) of the localization error of subject A in different walking styles.

In order to evaluate the applicability of our method in different ways of walking, subject A was asked to walk in four different ways (brisk walking with small and big steps, backward walking and quick walking). Subject A walked along the planned trajectory with each type of walking 10 times simultaneously, and the localization results were recorded. Figure 20 shows the obtained average localization trajectory of subject A walking with small steps over 10 repetitions of the experiment. Figure 21 gives the mean error curves and the standard deviation. An error bar graph is also presented to compare the indoor localization experiment results of the four types of walking, which is shown in Figure 22.

As shown in Figure 22, different ways of walking have different effects on the experimental results. It is observed that the localization error is biggest when the subject walked with a big step. Normally this type of walking makes the human body shake, which deteriorates the measurement error of our wearable sensors. Due to the limitation of wireless communication rates, the localization result is not satisfactory when the user is running. In contrast, when the pace is very small, the human body is then stable. Therefore, the smallest localization error is obtained among the four types of walking. In general, our method can be applied to different types of walking, the performance of the proposed algorithm is satisfactory compared with the existed methods proposed in [12,19].

## 5. Conclusions

This paper proposed an integrated wireless wearable sensor system that combines the functions of posture recognition and indoor localization. The developed low-cost sensor system has many advantages such as simple structure, light weight, small size, convenient maintenance and is very easy to use. The pitch angles of the left thigh and waist are used to recognize five human common postures. By calculating the coordinates of two hip joints, two knee joints and two ankle joints, the one-step vector can be obtained. Based on the one-step vector calculation and using the human body attitude information, the relative indoor localization is realized. The localization accuracy can be further improved by fusing the relative localization result and pre-setting the RFID tags’ positions using the set-membership filtering algorithm with incomplete observation. Experiments were conducted to verify the effectiveness of the proposed sensor system and corresponding algorithms.

It has to be pointed out that there are also some limitations in our sensor system. We can achieve very high positioning accuracy, but many sensors are needed. This may bring some inconvenience to users’ daily life. It also should be noted that the coordinates of six lower limb joints (two hip joints, two knee joints and two ankle joints) can be calculated by our system. These data are very useful for gait recognition and analysis in the field of rehabilitation. We would like to apply the proposed system in the field of lower limb rehabilitation for the elderly in the future.

## Figures and Tables

**Table 1:** The parameters of subjects.

Parameter	Subject A	Subject B	Subject C	Subject D	Description
$l_{h}$	73 cm	76 cm	75 cm	70 cm	The length of HAT(Head-Arm-Trunk)
$l_{waist}$	28 cm	30 cm	30 cm	32 cm	The distance between two hip joints
$l_{thigh}$	48 cm	52 cm	45 cm	50 cm	The length of thigh
$l_{shank}$	46 cm	50 cm	48 cm	48 cm	The length of shank

**Table 2:** The results of posture recognition.

Posture	Standing	Sitting	Squatting	Supine	Prone
Subject A	100%	100%	100%	100%	100%
Subject B	100%	100%	100%	100%	100%
Subject C	100%	100%	100%	100%	100%
Subject D	100%	100%	100%	100%	100%
